# Could Road Safety Education (RSE) Help Parents Protect Children? Examining Their Driving Crashes with Children on Board

**DOI:** 10.3390/ijerph18073611

**Published:** 2021-03-31

**Authors:** Francisco Alonso, Sergio A. Useche, Eliseo Valle, Cristina Esteban, Javier Gene-Morales

**Affiliations:** 1 DATS (Development and Advising in Traffic Safety) Research Group, INTRAS (Research Institute on Traffic and Road Safety), University of Valencia, 46022 Valencia, Spain; cristina.esteban@uv.es (C.E.); javier.gene@uv.es (J.G.-M.); 2 Department of Personality, Evaluation and Psychological Treatment, Faculty of Psychology, University of Valencia, 46010 Valencia, Spain; 3 Department of Education and School Management, University of Valencia, 46010 Valencia, Spain; jose.e.valle@uv.es; 4 PHES (Prevention and Health in Exercise and Sport) Research Group, University of Valencia, 46010 Valencia, Spain

**Keywords:** road safety education, parents, driving, road safety, risk behaviors, traffic crashes with children, children safety

## Abstract

Recent evidence suggests that driving behavior and traffic safety outcomes of parents may be influenced by the extent to which they receive information and education on road safety, as well as the fact of driving with their children on board, which may increase their risk perception. However, there are no studies specifically addressing the case of crashes suffered while driving with children. Hence, this study aimed to describe the relationship between road safety education-related variables and parents’ traffic safety outcomes while driving with children on board. For this cross-sectional study, data was retrieved from a sample composed of 165 Spanish parents—all of them licensed drivers—with a mean age of 45.3 years. Through binary logistic regression (logit) analysis, it was found that factors such as gender, having received road safety education (RSE), and having been sanctioned for the performance of risky driving behavior contribute to modulating the likelihood of suffering crashes while driving with children on board. Gender differences showed a riskier status for male parents. In this study, a set of risk factors explaining the involvement in traffic crashes when driving with children as passengers was identified among parents: gender, traffic sanctions, valuation, and exposure to road safety campaigns. Also, substantial limitations in the self-reported degree of received RSE were found, especially considering that risky driving behavior and traffic crash rates with children on board still have a high prevalence among parents.

## 1. Introduction

Road crashes involving children remain a major concern of public health, being the main cause of death among children between 15 and 19 years of age, as well as one of the main causes of death in age groups between 5 and 15 years [1]. This is especially relevant considering that most of these crashes involving children are caused by adult drivers [2]. Therefore, it is important to adopt a holistic perspective towards crash prevention, emphasizing all users who potentially partake in the problem. The key stakeholders involved in children’s road safety are mainly parents [3,4,5,6,7]. Although some studies have suggested that driving with children on board may contribute to decreasing the risk assumption of driving parents (also increasing their risk perception), most of the evidence agrees on the fact that more road safety education (RSE) is needed among them since it might improve parents’ skills for safe driving, or road safety skills [8,9,10].

The term “road safety skills” has been used in previous expert literature to refer to the gathering of a set of road-safety-friendly competencies acquired through road safety education (RSE), which are necessary for safe, healthy, harmonious traffic behavior [8,11]. Gender differences in road safety skills have been reported in some research, with a problematic divergence between men and women—males seem to obtain the poorest results in terms of positive attitudes towards road safety, traffic rule knowledge, and self-reported behaviors on the road [3,12]. Also, age is recognized as a potential influencing factor of road safety skills. Young adults tend to perform road misbehaviors according to certain characteristics and traits of their age group, such as impulsivity and sensation-seeking [13,14]. The lack of experience of young individuals makes them a highly vulnerable group, especially when they have only just obtained their driver’s license. Risky behavior and crashes on the road occur (in a considerable proportion) within the first months after obtaining the driver’s license; after this period driving performance improves [15] and risky behavior and crashes tend to drastically decrease [16]. Furthermore, the educational level may be a determinant of road safety skills and the existence of road crashes. While some authors found a significant positive correlation [17,18], others found no significant correlation between a drivers’ positive behavior and their educational levels [19,20,21,22].

Another key point on the prevention of road crashes resulting in injured children is road safety education or traffic safety education (RSE). Some studies argue in favor of the potential usefulness of RSE in preventing road crashes and promoting safe habits [8,23,24,25,26,27,28,29], although the outcomes seem to be dependent on the beneficiaries’ profile [30,31]. Other studies reported inconclusive results on the usefulness of RSE in improving road crash statistics [32,33,34,35,36,37,38]. Throughout the last decade, education in road safety has acquired a substantially important role in public health strategies aimed at strengthening the integrity and welfare of the entire population and improving prosocial behavior and convivence. According to the functional paradigm of road safety education (RSE), the prevention of risky behavior on the road—which is associated with a high number of traffic crashes—may be strengthened through the development of positive attitudes and perceptions, and accurate knowledge of traffic norms and safe behavior [39,40,41]. In the specific case of parents, road safety education constitutes a crucial issue, considering that they exert a relevant influence on the RSE of their children, who learn a significant part of their attitudes and behaviors on the road from their microsocial environment during the early stages of life [42,43]. Road safety education of parents plays, therefore, a relevant role in public health and within the sustainable development of countries [44] since it provides positive outcomes in the road safety skills of the community of drivers [3], and it may also condition the behaviors of future drivers [42,43]. In this sense, different stakeholders are involved in the development of road safety as a social issue, including schoolteachers, public and private institutions, and health agencies [23,30,40]. However, it is still necessary to develop further strategies aimed at increasing the actions related to road safety education and to reduce the objective road risk [3,45].

For these reasons, it could be very useful to provide more opportunities for adults, to allow them to update their knowledge through road training courses [46]. This would enable them to achieve a comprehensive knowledge and assessment of norms that systematically appear within the road traffic context. In addition, adults should always be aware of the importance of the measures adopted by the traffic authorities, such as increasing the presence of police, the improvement of infrastructures, and implementation of road safety campaigns. This would lead to fewer risks being taken and, at the same time, it would help children to learn safe attitudes towards driving. This is very important, since, as we have mentioned before, risky behavior performed by parents is usually repeated by their children [7]. Due to all the aforementioned facts, it is important to understand the factors associated with the learning of road safety, since this would help to prevent future risky behavior, traffic crashes (with and without children as passengers), and promote awareness and risk perception among children, which is also important.

### Objective and Hypotheses of the Study

The aim of this study was to describe the relationship between having received road safety education (recall of the contents, assessment of its utility and its importance), the importance attributed to different strategies aimed at improving road safety (improvement of roads and vehicles, police supervision, traffic fines), and road crashes with children on board suffered by Spanish adults. It was hypothesized that parents who had not received RSE and with lower awareness of road safety issues would have been involved in more road crashes.

## 2. Materials and Methods

### 2.1. Sample, Design, and Procedure

For this cross-sectional study, we used a total sample of *n =* 165 Spanish parents (78 men composing 47.3% of the sample, and 87 women, representing 52.7% of the sample) from 19 different provinces of Spain. With this sample size, a 0.98 power (1–ß) was achieved with an effect size (f^2^) of 0.15, assuming a 0.05 error. The mean age of parents participating in the study was *M* = 45.26 (*SD* = 6.19) years. All of them were active drivers of conventional automobiles, with a mean tenure as drivers of *M* = 8.42 (*SD* = 5.21) years since they had first obtained their driving license.

They were invited to participate in the study through the distribution of a national survey on road safety education, in which their children answered a set of questions in the classroom, and parents were also invited to voluntarily fill out a supplementary questionnaire on the same topic. They were asked to do so through a personal letter sent to them. Fulfilling an electronic informed consent form was necessary to access the e-survey. The global response rate (fully answered questionnaires) was approximately 35%, out of a total of 500 invitation letters initially delivered.

In order to ensure that the participants responded honestly to the survey, different strategies were adopted to minimize convenience in the answers. First, the survey was conducted guaranteeing the anonymity of participants and emphasizing the existing laws on data protection.

We also remarked that the collected information would only be used for statistical and research purposes and this was reported before the participants started to complete the questionnaire. The importance of answering honestly to all the questions was also emphasized, together with the non-existence of wrong or right answers. Participants were provided with written explanations in this regard, and emphasis was put on the study aim of gathering information about road safety-related experiences, perceptions, and practices, with the objective of developing scientific knowledge in this field.

### 2.2. Description of the Instrument

For this research, an electronic questionnaire composed of three dimensions (sociodemographic factors, road-safety-education-related factors, and driving specific information) was designed. The average time spent completing the questionnaire was *M* = 13.50 min. The first part, before commencing the questionnaire, was focused on the aspects that aimed at gathering honest data, such as the anonymity, non-existence of right/wrong answers, and the only-for-research-purpose statements. The sections were organized as follows.

First, a summary of sociodemographic information (i.e., age, gender, and educational level) and data related to road use (i.e., road crashes driving with children as passengers in the last five years, road crashes suffered as a passenger in the last five years, and traffic fines received while their children were on board), in order to characterize the participants according to their main features. Age was collected through an open question, while gender and educational level through a multiple-choice question including all possible options, such as male/female/other (gender), and none/primary school/high school/professional training/superior studies (education). Road crashes and traffic fines were collected as continuous variables.

A second section was used to assess the participation of parents in road safety education activities and all their related factors, such as the type of interventions, their duration, the value that participants attributed to them, and the scenarios employed for these interventions.

Finally, the third set of questions was designed to assess the parents’ perception of several factors related to road safety on a scale ranging from 0 (lowest value) to 10 (highest): assessment of measures aimed at the improvement of road safety (improvement of city roads and vehicles, and police supervision), assessment of the importance of road safety education, and perceived efficacy and utility of road safety campaigns.

### 2.3. Statistical Analysis (Data Processing)

Descriptive statistics (mean, standard deviation) was performed to present the global results and the associations among the study variables in our sample of Spanish parents. Spearman’s rho (bivariate) correlational analysis was used to look for associations between pairs of study variables, considering their robustness over Pearson’s (r) correlation when ordinal values are measured. Furthermore, gender comparisons were conducted using one-way analysis of variance (ANOVA) tests. Finally, after performing normality tests and testing basic statistical parameters, a logistic regression model was built to explain the odds ratio of having suffered one or more traffic crashes (as drivers, with children as a passenger) during the last five years (binomial dependent variable, in which 1 = Yes and 0 = No), using both demographic and RSE-related variables. The statistical analyses were performed using IBM SPSS (Statistical Package for Social Sciences), version 26.0 (IBM, Armonk, NY, USA) and the sample size calculation with the G*Power 3.1.9.6 (Heinrich Heine University Düsseldorf, Düsseldorf, Germany) [47].

## 3. Results

From the total sample of 165 parents, 78.2% had children who had previously participated in at least one program or intervention related to road safety education, organized by the educational center which they were attending. Almost 64% of the parents who participated in this research reported having suffered a traffic crash while driving with children during the previous five years (*M =* 1.50 *SD* = 1.78), and 45% had suffered a traffic crash as a passenger (*M =* 0.79 *SD* = 1.26). Furthermore, 29% of them had received at least one traffic ticket or fine driving with children during the same period. Regarding the coverage of RSE among Spanish parents, it was found that 85.4% of them reported having received some sort of program, course, or intervention related to road safety education throughout their lifetime.

### 3.1. Association between Sociodemographic Factors, Road Safety Education, Awareness of Road Safety Issues, and Driving Outcomes

The bivariate correlation analysis (see Table 1) allowed us to establish significant measures of association between the study variables related to both road safety education and the demographic profile of parents. Specifically, age was negatively and significantly associated with having received RSE, with older parents being the ones who tended to obtain lower values concerning this factor. On the other hand, age was positively associated with the perceived utility of RSE, with the degree of importance attributed to the improvement of vehicles as a way of enhancing road safety, and with the number of crashes suffered driving with children during the previous five years.

Regarding the educational level of parents, it was positively associated with the importance attributed to RSE, with the importance attributed to the improvement of city roads, and police supervision for the enhancement of road safety, and, finally, with the degree of efficacy perceived in the existing road safety campaigns. In other words, parents with higher education (greater educational level) tended to appreciate these factors less than parents with lower educational levels.

Finally, and specifically regarding road-safety-education-related variables, a set of significant measures of association (available more in detail in Table 1) was found between the following:

(a) Having received RSE, together with the importance attributed to it [+], to its improvement [+], and the perceived efficacy of road safety campaigns [+].
(b) The perceived utility of RSE in daily life and its attributed importance [+], the importance attributed to the improvement of city roads [+], the improvement of vehicles [+], and road safety education [+] as ways to improve road safety.
(c) The degree of effectiveness perceived in road safety campaigns and the agreement with the different mentioned strategies, used for improving road safety [+].

### 3.2. Gender Differences

When comparing the profile of parents and their scores in the different study variables related to RSE, a set of significant differences was found concerning the gender of respondents. A significant impact of gender was found in the fact of having a crash with onboard children, and in the perceived effectiveness of police supervision. To begin with, male parents presented a significantly higher mean value of crashes suffered as drivers with children on board during the previous five years (*M* = 1.85; *SD* = 1.92) than female parents (*M =* 1.18; *SD* = 1.58), with *F*_(1,163)_ = 6.041; *p* = 0.015.

Moreover, it was also found that women perceived the increase of police supervision as an alternative for improving road safety (*M =* 7.22; *SD* = 2.31) to a significantly higher degree than men (*M =* 6.42; *SD* = 2.06), with *F*_(1,163)_ = 5.429; *p* = 0.020.

### 3.3. Prediction of Self-Reported Road Crashes with Children on Board

This significant model, conducted through a binomial logistic regression technique, was fitted using both demographic and road safety and RSE-related variables. The function defining the binary logistic regression model is presented below:

*P*(*Y* = 1; *X*_*i*_) = *e*^*zi*^/1 + *e*^*zi*^ (1)

where: *z*_i_ = β_0_ + β_1_*X*_1_ + β_2_*X*_2_ + ··· β_10_*X*_10_ + ε

The variables included in the model were defined as:

- *Yi* = 1, implies having suffered at least one crash with children on board in the last 5 years.
- *Yi* = 0, means the driver did not suffer crashes with children on board during the same period.
- *X*_1_, *X*_2_… (…) …*X_n_*: Independent (whether continuous or categorical—Dummy) variables included in the model.

The final solution presented an overall accuracy percentage of 71.2%, explained 27.3% of the variance among subjects (Nagelkerke’s *R^2^* = 0.273), and showed a −2 Log-likelihood coefficient of 177.151. The basic parameters and variables included in the model are presented in Table 2, showing its Beta coefficients, significance level, and Confidence Intervals (CI) at 95%.

Overall, the logit model allowed it to be established that increased values in some of the variables contained in the model play a “protective” role, decreasing the Odds Ratio (OR) of belonging to Group 1 (i.e., having suffered a traffic crash in the last five years while driving with children on board). The variables considered in the final model were: having previously received road safety education (dummy), whose increase in one logarithmic unit (being positively responded) represents a decrease in 72.9% of the likelihood of belonging to Group 1 (crashed drivers); the number of traffic fines or penalties received, which decreases the OR by 71% per fine; and the degree of efficacy perceived in road safety campaigns, whose increase explains a decrease of the OR by 6.4% per logarithmic unit.

**Figure 1:**
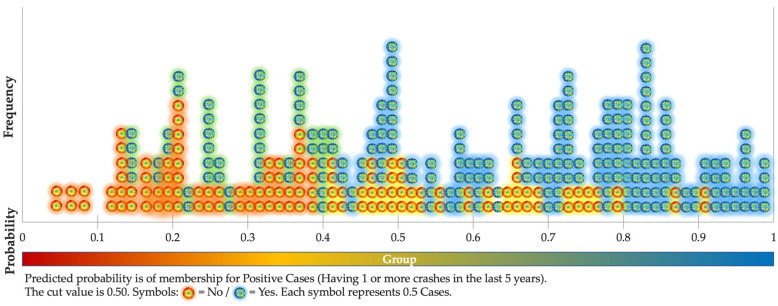
Observed groups (Logit) and predicted probabilities.

Figure 1 contains the predicted probabilities of the significant model, where (approximately) seven out of each 10 cases (71.2%) could be successfully predicted. The concentration of successfully predicted cases (blue/green spots in Figure 1) is predominantly located on the right-hand side of the distribution (i.e., “high probability profiles” that actually suffered crashes). On the other hand, most of the red/yellow spots (negative cases) are coherently distributed at the left-hand part of the figure, corresponding to these spots of “low probability profiles”, who actually did not suffer crashes while driving with children on-board.

## 4. Discussion

The results of this study support the existence of a relationship between factors related to road safety education and risky road behavior within a sample of Spanish parents driving with children. In order to accomplish the main purpose of the research, i.e., describing the relationship between road safety education (RSE) and traffic outcomes of parents, a significant regression model (Logit) was built.

Thanks to the reported logistic regression analysis, it is possible to state that road traffic crashes suffered when driving with children can be explained through factors related to behavior on the road, as well as through attitudes towards, and knowledge of, traffic norms, with a considerable amount of explained variance and a set of relevant correlations among study variables. Aiming at accounting for model uncertainty by averaging all plausible models [48,49], there are different methods such as the Bayesian Model Averaging (BMA) that can be useful in the analyses of sets of data such as the one presented in this study. While the traditional regression methods commonly select a single “true” model among a majority of alternative models based on some model selection criteria and may neglect the uncertainty related to the choice of models, BMA combines all plausible models (with various combinations of influential variables) using posterior probability as the weight [50,51]. BMA is an extension of the usual Bayesian inference methods that allow for direct model selection, combined estimation, and prediction [52]. Therefore, future studies using this approach with a similar study design are guaranteed. In brief, and in coherence with some other recent studies [27,53,54], some elements included in road safety education programs have been proven to modulate the risk of suffering a traffic accident. First, receiving RSE represents a substantial decrease in the OR of being involved in road crashes while driving with children as passengers (−72.9%). Second, a sanctions-related element is included in the model (i.e., having received traffic fines) that, in accordance with the aim of traffic fines [55,56,57], leads to an OR reduction (−71%) in the Logit model. Third, it is worth mentioning the value attributed to road safety campaigns, since being exposed to them implied an OR reduction (−6.4%). Finally, several relevant factors also increasingly influenced the OR: first, having suffered crashes as passengers, which represents a potential impairer of performance among road users, due to its psychological consequences such as anxiety, depression, and post-traumatic disorders [58,59,60]. In addition, and this appears to be even more relevant, being a male driver increases the chances of suffering a crash as well, if we take into account the prototypical risk factors of male drivers [61]. It is clear, and consistent with the evidence, that there is a need of developing more gender-focused interventions aimed at reducing risky road behavior among male drivers, considering (for instance) their general performance, driving style, aggressiveness, and other key elements that differentiate them from women [61,62,63,64].

Furthermore, the measures of association among study variables allowed us to establish some common points with the existing literature on road safety education, which, nevertheless, has been scarcely developed in Spain so far. First of all, the age of parents has been proven to be associated with lower participation in RSE. However, and coherently with the results shown by other empirical studies, older individuals tend to report a more positive assessment of road safety strategies and measures than young ones [23,65].

Regarding the parents’ role in the road formation of both their familiar and microsocial system, there is an empirically proven involvement of parents in both safety outcomes and road behaviors of their children [7,66]. In other words, empirical research has systematically demonstrated that parents have a crucial influence on their children’s learning of complex tasks such as safe driving and road risk prevention [5,67]. However, this phenomenon has some nuances that should be mentioned: in the same way as parents can exert a positive influence on their children’s road behaviors (i.e., contributing to the development of positive attitudes, beliefs, and behaviors that foster the safety of children), negative behavior and attitudes can also influence the way young road users perceive traffic safety and, subsequently, how they behave on the road [10,45].

Comparisons based on gender have shown that the self-reported risky behavior and the rates of suffered accidents (in this case, during the previous five years) have a significantly higher prevalence in men. In this regard, studies on risk perception argue that male adult drivers tend to perceive several road misbehaviors as generally less serious and less likely to result in a traffic crash, in addition to presenting a high explained variance in optimism and perceived invulnerability [68,69,70]. Glendon et al. [71] also found that there is a set of substantial differences in how drivers perceive their driving competencies and risk of crashes according to their gender, with males—especially younger ones—being the ones who consider themselves more often than anyone else to be better drivers, thus consequently misjudging the objective risk of their road misbehaviors. This explains their higher exposure to the performance of risky behavior and negative outcomes on the road.

Nevertheless, these results must be analyzed carefully, in the light of the key social changes that are currently happening within our society, such as the frequency with which women use vehicles and their involvement in different tasks related to driving (e.g., execution of transport-related jobs and the growing acquisition of driving licenses by female drivers). This is, perhaps, the factor resulting in their accident rates annually increasing in many countries, even though female-involved crashes are still lower than men’s road causalities [72,73]. In this sense and resuming the discussion about the influence of parents on how young individuals learn to behave in the road environment, it is foreseeable that there will be a subsequent increase in the influence of mothers on children’s road behavior over the following years, considering that, in addition to being involved as key stakeholders per excellence in their children’s safety education, they are also progressively becoming an observational reference of road behavior for them.

Finally, it is worth discussing the need to improve and strengthen parental strategies aimed at the road safety education of children, based on three particular facts observed in this study: first, the current relatively low coverage of RSE in parents. Even though 85.4% of parents stated that they had participated in educational programs on road safety before the data collection of this research, we should consider that their main source of information was to be found in driving schools (which focus their teaching almost exclusively on road formation, leaving road safety education aside). In fact, the most successful interventions within RSE are usually associated with its integration into the educational system and with the involvement of family members [3,40]. Also, even in the most developed countries, there is still a clear lack of interventions aimed at improving parental strategies that ensure their children’s safe behavior on the road, and this could be a relevant innovation if it were implemented in Spain [3]. In concluding, it is important to remark on the existing need to involve parents in actions promoted by schools, aimed at strengthening the children’s RSE learning, as it has been recently suggested in some other empirical experiences [10,31,65].

### Limitations of the Study

Although all statistical parameters were accurately and positively tested during the data treatment, some specific issues present in this research should be listed as potential biasing sources. Specifically, one of the most important limitations of this work is related to the use of self-reports as our primary source of information, and this could be associated with biases such as social desirability [74], or poor understanding of the questions, especially considering that a significant number in the sample had no clear understanding of the concept of “road safety education”. It also would be necessary to consider random heterogeneity within the variable effect, as stated by previous research [75,76,77]. Regarding this, and as a recommendation for future research, it is important to suggest the use of supplementary measures for the assessment of road safety behavior, accounting for age and gender, whose greatest asset could be, in this case, the minimization of the “common method biases” that often affect cross-sectional designs [78].

Additionally, other useful methods, apart from descriptive analyses (such as modeling by structural equations; [79], may allow researchers to statistically explain the parents’ perceptions of the issues addressed in the study. Another statistical method that may improve the prediction performance and account for heterogeneity in the sample (see [80,81] for further information) could be Bayesian Modelling Averaging (BMA). Furthermore, some variables that were not evaluated in this study [82], such as the rates of road crashes and the attitudes towards road safety, could be useful for deepening the discussion of the results in the light of the individual characteristics of road users [83]. Finally, the study sample is still relatively small (although the power analysis did provide appropriate values), which makes it difficult to give high external validity to this research. Nevertheless, all statistical analyses were conducted in the light of the fulfillment of basic parameters, needed to perform statistical tests. Other sample size evaluation criteria were used as shown in previous research [84,85].

## 5. Conclusions

To sum up the findings of this study, a statistical association between road safety education indicators and the road traffic crashes suffered while driving with children on board reported by parents was established. Furthermore, we identified different risk factors that increase the probability of suffering traffic crashes while driving with children: gender, traffic sanctions, valuation, and exposure to road safety campaigns. Finally, it is important to highlight the need for interventions aimed at improving parental road safety education, and their influence on children’s behavioral outcomes on the road.

### Practical Applications

- The outcomes reported in this study highlight the need to improve road safety education (that is predominantly given to children) also among parents.
- Although all drivers deserve attention (all of them have certain risk factors attached), this study has found that older males have a greater likelihood to suffer crashes while driving with their children on board.
- Road safety campaigns remain a useful tool for developing positive driving outcomes among parents, especially whenever these communicative actions are positively valued by them.
- Although it is known that traffic sanctions have a limited corrective effect, drivers receiving traffic fines could be less likely to have a crash with onboard children, supporting the value of traffic law enforcement on traffic crash prevention.

## Figures and Tables

**Table 1:** Bivariate (Spearman) correlations between study variables.

	Variable	2	3	4	5	6	7	8	9	10	11	12	13	14
1	Age	0.015	−0.172 *	0.137	0.159 *	0.008	0.066	0.184 *	−0.144	0.004	0.128	0.123	0.236 **	0.099
2	Educational level	−	−0.078	−0.094	−0.103	−0.242 **	−0.174 *	0.028	−0.237 **	−0.138	−0.179 *	0.026	−0.040	0.073
3	Have you ever received road safety education?		−	0.219 **	0.227 **	−0.005	−0.108	−0.109	0.203 **	0.153 *	0.013	−0.013	0.058	−0.037
4	Recalling of road safety education contents			−	0.790 **	0.221 **	0.293 **	0.153	−0.035	0.271 **	0.117	−0.039	−0.008	−0.048
5	Perceived utility of road safety education				−	0.324 **	0.374 **	0.209 **	0.034	0.433 **	0.295 **	0.016	−0.068	0.012
6	Importance attributed to road safety education					−	0.415 **	0.283 **	0.229 **	0.600 **	0.243 **	−0.03	0.025	0.067
7	Importance attributed to the improvement of city roads						−	0.493 **	0.170 *	0.417 **	0.283 **	0.123	0.054	−0.119
8	Importance attributed to the improvement of vehicles							−	0.178 *	0.311 **	0.201 *	−0.102	0.020	0.060
9	Importance attributed to the improvement of police supervision								−	0.246 **	0.222 **	−0.151	−0.128	−0.105
10	Importance attributed to the improvement of road safety education									−	0.203 **	0.019	−0.013	0.075
11	Perceived efficacy of road safety campaigns										−	−0.117	−0.090	0.007
12	Crashes suffered as a passenger (5 years)											−	0.405 **	0.074
13	Crashes suffered as a driver with children on board (5 years)												−	0.227 **
14	Have you ever been fined with children on board? (5 years)													−

Notes for the table: * Correlation is significant at *p* < 0.05 level. ** Correlation is significant at *p* < 0.01 level.

**Table 2:** Logistic regression model (logit) to explain traffic crashes suffered by parents while driving with children.

Variables in the Equation	B	S.E.	Wald	df	Sig.	Exp(B)	CI (95%) Exp(B)	
							Lower	Upper
Gender (Man ^a^)	0.951	0.379	6.287	1	0.012 *	2.589	1.231	5.444
Have you ever received road safety education (Yes ^b^)?	−1.304	0.516	6.397	1	0.011 *	0.271	0.099	0.746
Traffic fines received with children on board (5 years)	−1.236	0.458	7.295	1	0.007 **	0.290	0.118	0.712
Crashes suffered as passenger (5 years)	0.663	0.232	8.17	1	0.004 **	1.940	1.232	3.056
Perceived efficacy of road safety campaigns	−0.066	0.033	3.983	1	0.046 *	0.936	0.877	0.999
Constant	2.207	0.858	6.621	1	0.010 **	9.093		

Dependent variable: Having suffered driving crashes with children on board in the previous 5 years (positive cases). Notes for the table: ^a,b^ Dummy variables. Success categories = ^a^ Being a male, ^b^ Having received road safety education (RSE); ** Significant at the level *p* < 0.01; * Significant at the level *p* < 0.05.

## Data Availability

The data that support the findings of this study are available from the corresponding author, upon reasonable request.
